# Co-expression and prognostic significance of putative CSC markers CD44, CD133, wild-type EGFR and EGFRvIII in metastatic colorectal cancer

**DOI:** 10.18632/oncotarget.26722

**Published:** 2019-03-01

**Authors:** Said Abdullah Khelwatty, Sharadah Essapen, Izhar Bagwan, Margaret Green, Alan M. Seddon, Helmout Modjtahedi

**Affiliations:** ^1^ School of Life Sciences, Pharmacy and Chemistry, Kingston University London, Kingston, UK; ^2^ St. Luke's Cancer Centre, Royal Surrey County Hospital, Guildford, Surrey, UK; ^3^ Department of Histopathology, Royal Surrey County Hospital, Guildford, Surrey, UK

**Keywords:** CSC, colorectal cancer, EGFR, immunohistochemistry

## Abstract

The presence of colorectal cancer stem cells (CSCs) have been associated with tumour initiation and resistance to therapy. This study investigated the co-expression and prognostic significance of the CSCs biomarkers CD44 and CD133 with wild-type EGFR (wtEGFR) and EGFRvIII in colorectal cancer (CRC). The expression of these biomarkers were determined in tumours from 70 patients with metastatic CRC by immunohistochemistry, and in a panel of human CRC cell lines, and their variants with acquired-resistance to EGFR inhibitors, by flow cytometry. The expression of CD44, CD133, wtEGFR and EGFRvIII were present in 17%, 23%, 26% and 13% of cases and the co-expression of CD44/CD133 with wtEGFR and EGFRvIII were present in 9% and 3% of the cases respectively. Only co-expression of CSCs/EGFRvIII (*P* = 0.037), and amphiregulin (*P* = 0.017) were associated with worse overall survival. Interestingly, disease-free survival was improved in BTC expressing patients (*P* = 0.025). *In vitro* CD133 expression and its co-expression with CD44 were associated with primary-resistance to irinotecan and acquired-resistance to anti-EGFR inhibitors respectively. Our results suggest co-expression of CSCs and EGFRvIII could be potential biomarkers of worse overall survival and resistance to therapy in patients with mCRC and warrants further validation in a larger cohort.

## INTRODUCTION

Colorectal cancer (CRC) remains ones of the leading causes of cancer deaths worldwide. Currently, CRC is estimated to be the third most commonly diagnosed cancer (140,250) and the third leading cause of cancer deaths (50,630) in the USA [1], highlighting the need for the development of more effective and less toxic therapeutic agents. The traditional model of tumourigenesis has been based on the idea that every cell within the tumour population is capable of tumour initiation and propagation. However, in the last decade with the emergence of the cancer stem cell (CSCs) hypothesis, it is postulated that a small fraction of cells are capable of tumour initiation and propagation and their presence are associated with a more aggressive tumour type [2–8]. Colorectal CSCs are characterised by the expression of CD133 positive cells [2, 4] and increasingly other surface markers such as CD44, Lgr5, EpCAM have been included in this profile [9–17].

Since the early 1980s, aberrant expression of the epidermal growth factor receptor (EGFR) has been widely reported in a range of epithelial malignancies including colorectal cancer. As such the EGFR remains an important therapeutic target for therapy with anti-EGFR monoclonal antibodies (mAbs) cetuximab and panitumumab, which have been incorporated into treatment paradigms for patients with refractory metastatic CRC [18–22]. However, a major challenge is intrinsic drug-resistance and/or acquired-resistance following a short course of therapy in patients with CRC. Indeed, RAS mutations have served as an important negative predictive biomarker for the primary resistance to therapy with anti-EGFR mAbs in patients. In another study, the CD133 was found to be increased in CRCs that are hyper-activated by mutations in the RAS-RAF-MEK-ERK pathways (Kemper et al., 2012). However, not all patients with wild type *KRAS* respond to therapy with anti-EGFR mAbs [23, 24] and objective responses of up to 44% have been reported in mCRC patients with *KRAS* mutations treated with FOLFIRI plus cetuximab in other studies [25].

To our knowledge, there are currently no studies on the co-expression and prognostic value of the putative CSCs biomarkers CD44, CD133, the wtEGFR and its heterologous ligands, and the type III-EGFR mutant (i.e. EGFRvIII) in patients with mCRC. Therefore, in this study using specific antibodies, we investigated the prognostic value of the co-expression of CD44, CD133, EGFRvIII, wtEGFR, and EGFR ligands in tumour specimens from 70 mCRC patients. We also investigated the expression levels of CD44, CD133 in a large panel of CRC cell lines and their association with response to treatment with standard cytotoxic drugs and the EGFR inhibitors. Moreover, using CRC cells and their drug-resistant variants, we investigated the role of CD44 and CD133 in the development of acquired-resistance to the EGFR inhibitors.

## RESULTS

### Clinicopathological features

Patient clinicopathological characteristics are summarised in Table 1. The median patient follow-up time was 4 years. None of the patients had received radiotherapy or chemotherapy prior to surgery. Forty three patients received post-operative adjuvant chemotherapy. Patients with tumours of N2 stage were found to have a shorter overall survival (*P* = 0.004) and disease-free survival (*P* = 0.0003). No significant association was found between survival and the other prognostic factors (Table 1).

**Table 1:** Patient clinicopathological characteristics and their association with overall survival and disease free survival using Kaplan-Meier analysis and log-rank Chi-squared test in 70 metastatic colorectal cancer patients

Characteristics	*N*	OS in years (mean ± SE)	95% CI	*P*-value (*χ*^2^-test)	DFS in months (mean ± SE)	95% CI	*P*-value (*χ*^2^-test)
**Age in years**							
≤70	19	5.478 ± 0.527	4.446–6.511	NS	58.737 ± 8.250	42.567–74.907	NS
>70	51	5.785 ± 0.361	5.078–6.492		81.460 ± 6.288	69.136–93.785	
**Gender**							
Male	40	5.930 ± 0.415	5.117–6.743	NS	78.636 ± 7.630	63.682–93.590	NS
Female	30	5.476 ± 0.450	4.593–6.359		75.558 ± 8.212	59.464–91.653	
**Tumour Site**							
Right colon	35	5.600 ± 0.449	4.720–6.480	NS	78.968 ± 8.184	62.927–95.008	NS
Left colon	35	5.843 ± 0.419	5.022–6.663		77.017 ± 7.269	62.770–91.265	
**T stage**							
T4	18	5.477 ± 0.695	4.116–6.839	NS	71.000 ± 9.247	52.876–89.124	NS
<T4	52	5.783 ± 0.342	5.113–6.453		76.092 ± 6.519	63.315–88.868	
**N stage**							
N2	41	3.971 ± 0.308	3.368–4.574	0.004	36.489 ± 4.702	27.273–45.706	0.0003
<N2	19	6.047 ± 0.329	5.401–6.693		88.842 ± 5.839	77.397–100.286	
**M stage**							
M0	62	5.832 ± 0.315	5.214–6.450	NS	79.917 ± 5.951	68.252–91.581	NS
M1	8	4.208 ± 0.601	3.031–5.386		45.429 ± 6.432	32.822–58.035	
**LVI**							
Absent	48	5.949 ± 0.349	5.265–6.632	NS	82.441 ± 6.933	68.851–96.030	NS
Present	22	5.142 ± 0.568	4.029–6.255		57.516 ± 6.191	45.381–69.650	
**Grade**							
G3	41	5.869 ± 0.464	4.960–6.779	NS	74.818 ± 7.324	60.463–89.173	NS
<G3	29	5.592 ± 0.408	4.792–6.392		79.061 ± 8.776	61.860–96.262	
**Chemotherapy**							
No	17	5.250 ± 0.618	4.040–6.460	NS	58.063 ± 8.071	42.244–73.881	NS
Yes	43	6.012 ± 0.450	5.257–6.766		80.511 ± 6.888	67.010–94.011	

### Immunohistochemical expression of CD44, CD133, EGFRvIII, wtEGFR and ligands in colorectal tumors

**Figure 1:**
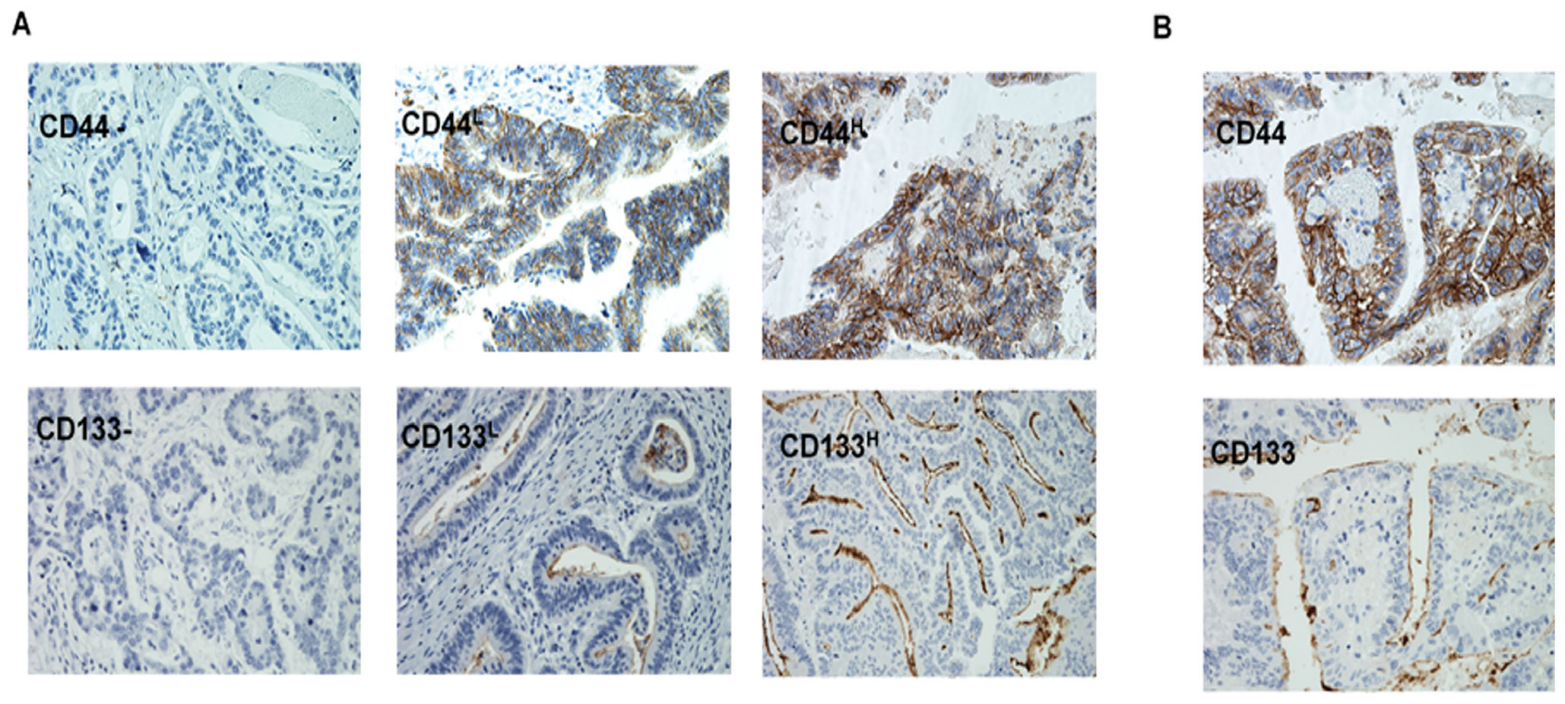
Immunohistochemical staining of the metastatic colorectal cancer specimens for CD44 and CD133 expression showing the intensity of staining (**A**), and the cellular location of staining (**B**).

The expression of CD44 and CD133 was determined in all 70 Dukes’ C and D colorectal cases by immunohistochemistry. The pattern of CD44 immunostaining was found to be membranous and low and high expression in 83% and 17% of the patients respectively (Figure 1A and Table 2A). CD133 was found to stain the luminal border with immunostaining present in necrotic debris in well differentiated tumours and a dot-like staining in poorly differentiated tumours with 23% and 77% of the cases expressing high and low levels of CD133 staining (Figure 1B and Table 2A).

Table 2Immunohistochemical expression of (A) CD44, CD133, (B) wtEGFR, EGFRvIII and EGFR ligands and their co-expressions in 70 metastatic colorectal cancer patients

**Table:** 

A					
Variables	No. of positive tumours (%)				
	% Positive tumour cells		Location		
	0–50 (Low)	>50 (High)	Mem	Cyto	Luminal
**CD44**	58 (83)	12 (17)	70 (100)	-	-
**CD133**	54 (77)	16 (23)	-	-	70 (100)

Abbreviations: Mem, Membranous; Cyt, Cytoplasmic.

**Table:** 

B									
Variables	No. of positive tumours (%)								
	% Positive tumour cells				Intensity			Location	
	>5	>10	>20	>50	1+	2+	3+	Mem	Cyto
**wtEGFR**	18 (26)	12 (17)	9 (13)	4 (6)	15 (21)	2 (3)	1 (1)	17 (24)	2 (3)
**EGFRvIII**	9 (13)	5 (7)	4 (6)	4 (6)	9 (13)	-	-	-	9 (13)
**Amphiregulin**	35 (50)	4 (6)	11 (16)	20 (29)	26 (37)	14 (20)	-	-	35 (50)
**BTC**	53 (76)	3 (4)	14 (20)	40 (57)	45 (64)	25 (36)	-	-	53 (76)
**EGF**	49 (70)	6 (9)	5 (7)	34 (49)	36 (51)	25 (36)	-	-	49 (70)
**TGFα**	51 (73)	2 (3)	7 (10)	39 (56)	47 (67)	4 (6)	-	-	51 (73)
**wtEGFR/1 ligand**	11 (16)	-	-	-	-	-	-	-	-
**wtEGFR/1 or more ligands**	17 (24)	-	-	-	-	-	-	-	-
**Any CSC/wtEGFR**	6 (9)	-	-	-	-	-	-	-	-
**Any CSC/EGFRvIII**	2 (3)	-	-	-	-	-	-	-	-

Abbreviations: Mem, Membranous; Cyt, Cytoplasmic.

**Figure 2:**
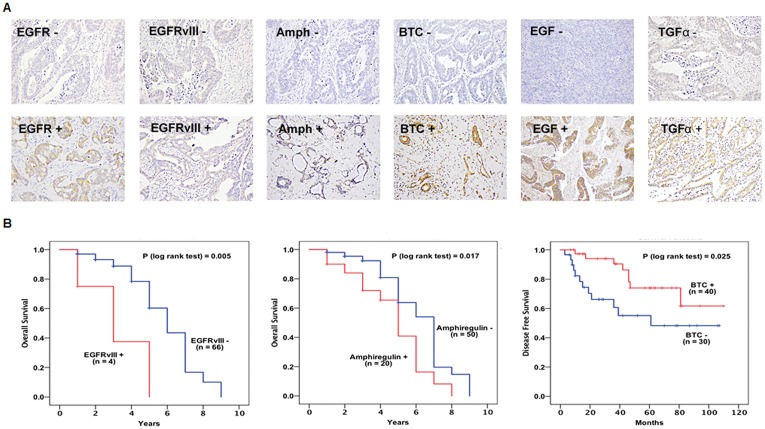
Immunohistochemical expression of wtEGFR, EGFRvIII, and EGFR ligands (**A**), association between the expression of wtEGFR, amphiregulin and BTC and survival shown with Kaplan Meier curves (**B**) in 70 metastatic colorectal cancer patients.

At cut-off value of >5%, wtEGFR expression was membranous in 26%, while EGFRvIII expression was observed predominantly in cytoplasm in 13% of the colorectal patients (Figure 2A and Table 2B). Of the EGFR ligands, amphiregulin, BTC, EGF, and TGFα were expressed in the cytoplasm of 50%, 76%, 70%, and 73% of the tumours respectively (Figure 2A and Table 2B). Immunohistochemical expression of epiregulin, HBEGF, and epigen were undetectable in this study.

Of the 70 CRC patients examined, 36% expressed at least one of the CSC markers (CD44 or CD133). However, the co-expression of CD44 or CD133 with EGFRvIII and wtEGFR was found in only 3% and 9%, respectively. At cut-off value >5%, 17/70 patients were found to co-express wtEGFR with at least one ligand while 11/70 patients co-expressed wtEGFR with all 4 ligands (Table 2B).

### CD133, EGF and TGFα expression is significantly associated with clinicopathological parameters

The expression of CSCs, EGFRvIII, wtEGFR and its ligands was analysed against clinicopathological characteristics of patients using Chi-squared and Fisher Exact tests. A significant association was found between the expression of CD133^H^ and tumours with <G3 differentiation (*P* = 0.019). At cut-off value >50%, the expression of TGFα was also significantly associated with tumours <G3 (*P* = 0.028). Interestingly, EGF expression above a cut-off value of 50% was significantly associated with M1 stage (*P* = 0.002).

### EGFRvIII, amphiregulin, and BTC is significantly associated with survival

A significant association was found between EGFRvIII (*P* = 0.005) and amphiregulin (*P* = 0.017) expressions at cut-off value of >50% and shorter overall survival (Figure 2B). Univariate analysis found a 4.5 fold and 2 fold increased risk of a shorter overall survival with expression of EGFRvIII (*P* = 0.016) and amphiregulin (*P* = 0.04), respectively and remained independent prognostic indicators of survival when analysed in multivariate analysis in this study (Table 3).

**Table 3:** The association of expression of EGFRvIII, amphiregulin with overall survival (OS) and BTC with disease-free survival in 70 metastatic colorectal cancer patients using multivariate Cox regression analysis

Overall Survival (OS)						
Variables	Univariate			Multivariate		
	HR	95% CI	P-value	HR	95% CI	P-value
**EGFRvIII**	4.568	1.325–15.748	*0.016*	7.215	1.941–26.817	*0.003*
**Amphiregulin**	2.070	1.035–4.139	*0.04*	2.082	1.040–4.167	*0.038*
**Disease-free Survival (DFS)**						
**Variables**	**Univariate**			**Multivariate**		
	**HR**	**95% CI**	***P*-*value***	**HR**	**95% CI**	***P*-*value***
**BTC**	0.375	0.153–0.920	*0.032*	0.369	0.150–0.910	*0.030*

*P*-value of ≤0.05 was considered significant.

The expression of BTC at cut-off value of >50% was found to be significantly associated with longer disease-free survival (*P* = 0.025) (Figure 2B) and multivariable analyses showed that BTC expression was an independent prognostic indicator of favourable disease-free survival (HR = 0.369, CI = 0.150 – 0.910, *P* = 0.03) in these patients (Figure 2B and Table 3).

Interestingly, the co-expression of CD44 or CD133 with EGFRvIII was significantly associated with shorter overall survival (*P* = 0.037) and remained an independent prognostic indicator of overall survival when adjusted for multivariable effect (HR = 5.451, CI = 1.193 – 24.906, *P* = 0.029) (Table 3).

### CD44 and CD133 expression in human colorectal tumor cell lines

**Figure 3:**
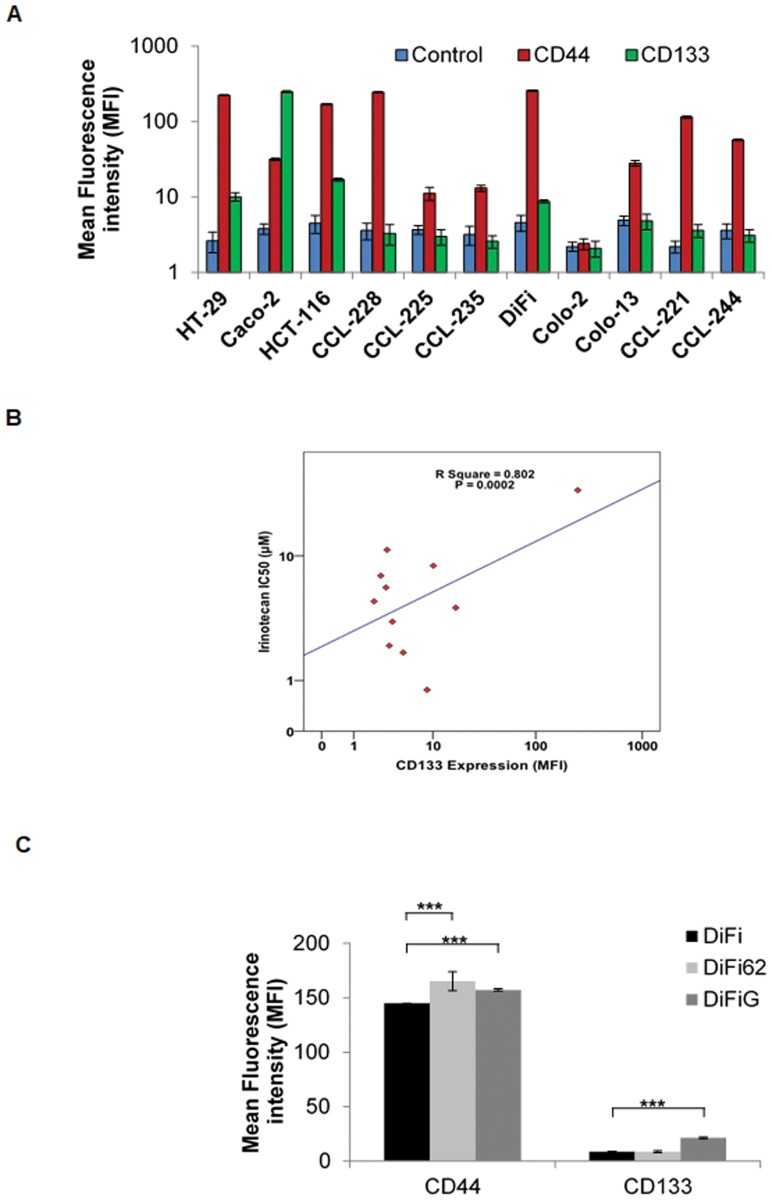
Expression of CD44 and CD133 in human colorectal tumour cell lines (**A**), association between CD133 expression and treatment with irinotecan (**B**), expression of CD44 and CD133 in DiFi parental versus DiFi62 and DiFiG acquired resistant variant cells (**C**), ±SD determined by flow cytometery (*n* = 3).

The cell surface expression of CD44 and CD133 was determined by flow cytometry in reference to positive control cell lines (Figure 3A). Of the human colorectal tumour cell lines examined in this study, HCT116, HT29, CCL-228 and DiFi cells were highly CD44 positive (i.e. >95% of tumour cell populations), while CCL-225 and Colo-2 cells were CD44 negative (Figure 3A). CD133 positive cell population was much lower in the majority of colorectal tumour cells, with only CaCo-2 cells expressing CD133 in more than 95% of the cells (Figure 3A).

### Association between expression of CD44 and CD133 and response to treatment with cytotoxic drugs

We have reported previously the growth response of human colorectal tumour cell lines to treatment with anti-EGFR mAb ICR62 and cytotoxic drugs [26]. Of the cell lines examined, only the EGFR overexpressing DiFi cells were highly sensitive to treatment with anti-EGFR mAb ICR62. We found no significant association between the expression of CD44 and/or CD133 and response to treatment with anti-EGFR mAb ICR62, irreversible ErbB inhibitor afatinib, and reversible ErbB inhibitors erlotinib and gefitinib (data not shown). Of the cytotoxic drugs, no significant association was found between the expression of CD44 and/or CD133 and response to treatment with 5FU or Oxaliplatin. Interestingly, there was a significant association between the expression of CD133, and its co-expression with CD44 and resistance to treatment with irinotecan (Figure 3B).

We reported previously development of the EGFR overexpressing DiFi cell variants with acquired resistance to the anti-EGFR mAb ICR62, and TKI gefitinib [27]. Interestingly, we found the expression of CD44 to be significantly increased in both resistant variants DiFi62 and DiFiG cells (Figure 3C). CD133 expression, on the other hand was significantly increased only in DiFiG cells.

## DISCUSSION

CSC are characterised by slow proliferation and cell cycle and as such have been associated with resistance to anticancer therapies through the deployment of various defence mechanisms such as activation of anti-apoptotic proteins, efflux pumps, and quiescence [28]. Because conventional chemotherapies target rapidly growing cells, initial treatment response in the form of tumour shrinkage is seen but they fail to eradicate the cancer as the tumour initiating CSCs are spared.

In this study, we have evaluated the association between the co-expression of putative CSC markers CD44, CD133, which have been shown to identify cells with tumour initiating and progression properties [3–7, 29]. Our findings suggest that despite putative CSC markers CD44 and CD133 being expressed commonly and the expression of CD133 being associated significantly with well and moderately differentiated tumours, there was no significant association with patient outcome in this study. Indeed, the prognostic significance of CD44 and CD133 in patients with CRC has been investigated in several studies [11, 30–33]. However, to our knowledge only four studies have investigated the combined expression and prognostic significance of both CD44 and CD133 in CRC patients using IHC and found contradicting results. Horst and colleagues investigated the expression of CD44, CD133 and CD166 in 110 CRC patient tumours by IHC and found 33%, 52% and 64% of the cases positive respectively. The co-expression of all three markers was found in 36% of the cases and concluded CD133 to be the best sole marker to predict low patient survival [32]. Another study, which investigated the expression of CD24, CD44 and CD133 in 523 tissue microarrays from CRC patients by IHC, found CD24, CD44 and CD133 expression in 51%, 96% and 25% of the cases examined respectively. Although the study found CD133 expression to be associated with gender and advance T-stage, no significant association was found between expression of CD44 and CD133 and survival [30]. Furthermore, Lugli and colleagues using TMA evaluated 1420 primary CRC and 57 normal mucosa samples for the expression of CD133, CD44s, CD166, EpCAM, and ALDH1 but failed to find any association between expression of these markers and patient outcome [34]. More recently, another study conducted by Jing *et al.* (2015) examined 36 colorectal primary adenocarcinomas with synchronous hepatic metastasis and found CD44, but not CD133, expression was independent factor associated with survival [35]. Finally, the results of two recent meta-analyses of 65 studies have also indicated that the high expression of CD133 is associated with poorer 5year overall survival and disease-free survival highlighting that CD133 could serve as a predictive biomarker of poor prognosis and treatment failure as well as a potential therapeutic target in patients with mCRC [36, 37].

One of the findings of the present study was that the co-expression of CSCs and EGFRvIII was found to be an independent factor associated with worse overall survival, albeit in a small number of patients. EGFRvIII is a ligand-independent, mutated form of the EGFR and its expression has been associated with poor prognosis in several human cancers [38–40]. To our knowledge, there have been no other studies on the co-expression of CSCs and EGFRvIII, wtEGFR and its ligands in patients with mCRC and as such this is a novel finding and warrants further investigation in a larger cohort of mCRC patients. The aberrant expression of EGFR and its ligands have been associated with poor outcome to therapy in various human malignancies, including CRC [38, 41–44]. In the present study while we did not find any association between the wtEGFR expression and survival, expression of amphiregulin was found to be a significant independent factor for poorer overall survival in these patients, which was consistent with the findings of other studies [45–49]. Interestingly, we found BTC expression to be a significantly associated with a favourable outcome in patients with mCRC. However, more recently, another study conducted by Yun *et al.* that examined 331 CRC for the association between the expression of EGFR family members and its ligands. While the study did not find any association between BTC expression and overall survival, they found the expression of HER4, another member of the EGFR family, to have a favourable outcome in patients with CRC [41]. BTC is known to also bind to HER4, which has been shown to be associated with better outcome in some cancers, including breast and gastric [50–52]. Indeed, the expression of HER4 although not determined in the present study, was determined in our previous study on the same cohort of patients with high expressions of HER4 (data not shown) [53]. This could, in part, provide an explanation for the association of BTC expression interacting with the overexpressed HER4 in these patients and contributing to a longer disease-free survival in the present study.

Nautiyal *et al.* investigated EGFR regulation of colon cancer stem-like cells (CD44, CD166 and ALDH-1 positive cells) and found EGFR inhibition by anti-EGFR mAb cetuximab diminished age-related increase of CD166 and ALDH-1 suggesting EGFR could have an important role in the regulation of colorectal CSC [54]. In this study, for the first time we investigated the expression of CD44 and CD133 in a large panel of human colorectal tumour cell lines and determine association to sensitivity to treatment with anti-HER inhibitors and cytotoxic drugs. We found that CD44 expression is more common than CD133 expression in human colorectal tumour cell lines and patient samples. While we did not find any significant association between expression of CD44 and/or CD133 and response to anti-EGFR mAb ICR62, and irreversible erbB inhibitor afatinib, reversible small molecule tyrosine kinase inhibitors erlotinib and gefitinib (data not shown), there was a significant association between CD133 expression and its co-expression with CD44 and response to treatment with the cytotoxic drug irinotecan. These findings are in line with previous studies of other cancer types where the expression of CSCs were found to be associated with radio- and/or chemo-resistance in cancers of prostate, hepatocellular and breast [55–58]. In addition, we have also observed a significant increase in the expression of CD44 and CD133 in CRC tumour cells with acquired resistance to anti-EGFR mAb and TKI therapies further highlighting the role played by CSCs not only in primary drug resistance, but also in the development of acquired drug resistance. However, further studies are warranted and should confirm the possible link between expressions of CSCs and response to treatment with the EGFR inhibitors.

In conclusion, our results suggest that co-expression of CSCs and EGFRvIII and the expression of amphiregulin are associated with poorer overall survival in patients with mCRC. However, expression of other ligands such as BTC, through its interactions with other EGFR family members such as HER4, could contribute to a longer disease-free survival in some mCRC patients. Since colorectal cancer was predicted to be the third most commonly diagnosed cancer and the second leading cause of cancer deaths worldwide in 2018 [59], our results support the need for further studies and its validation on the prognostic significance of the co-expression of CSCs, EGFRvIII, and the wtEGFR and its ligand and their predictive value for the response to therapeutic interventions in a larger group of patients with mCRC.

## MATERIALS AND METHODS

### Tumor cell lines

The human colorectal tumour cell lines CCL-221 (Dukes’ C), CCL-225 (Dukes’ C), CCL-228 (Dukes’ B), CCL-244 (tumour stage unknown) and CCL-235 (Dukes’ D), CaCo-2 were purchased from the American Type Culture Collection (Manassas, VA) and HCT-116 (tumour stage unknown), HT-29 from European Collection of Cell Culture (Porton Down, United Kingdom). Other human colorectal tumour cell lines used in this study included our two cell lines Colo-2 (Dukes’ A) and Colo-13 (Dukes’ C) [60] and the EGFR over-expressing cell line DiFi which was established from a patient with familial adenomatous polyposis (FAP) and was kindly provided by Dr Z Fan (MD-Anderson Cancer Centre). Acquired drug resistant variants of DiFi cells were developed as described previously [27]. All cell lines were routinely cultured in Dulbecco's modified Eagle's medium (DMEM) (Merck, Gillingham, UK) supplemented with 10% foetal bovine serum (FBS) (Merck, UK) and the antibiotics penicillin, streptomycin and neomycin and were maintained at 37°C in a humidified atmosphere with 5% CO_2_.

### Antibodies, EGFR inhibitors and other reagents

The rat monoclonal antibody ICR62 (IgG_2b_) was raised against the external domain of the EGFR on the breast carcinoma cell line (MDA-MB468) [61]. The secondary antibody used in this study included FITC-conjugated rabbit anti-mouse IgG Star 9B (Bio-rad Antibodies, Kidlington, UK). The cytotoxic drugs, 5-FU, Irinotecan and Oxaliplatin were purchased from Merck, UK.

### Flow cytometry

The cell surface expression level of CSC markers CD44 and CD133 in human colorectal tumour cell lines and drug resistant variants was determined using FACScalibur, employed for the determination of EGFR family members (Becton Dickinson, Oxford, UK) as described previously [26].

### Patient information

Seventy patients with metastatic CRC (Dukes’ C and D) who underwent radical surgery at the Royal Surrey County Hospital (Guildford, UK) between April 2002 and November 2007 were included in this retrospective study. Ethical approval was obtained from the Research and Development Committee of the Royal Surrey County Hospital. Those with no follow-up information, mis-diagnosis, and incomplete histology were excluded. Cases of peri- and post-operative death (i.e. within 6 months of surgery) were also excluded from this study, as were those with tumour blocks in a condition too poor for immunohistochemical use. Detailed clinicopathological information, including patient age and gender was available for each patient.

### Immunohistochemistry

Paraffin-embedded sections of tumour specimens (3 μM) and control cell pellets (Caco-2) were cut from paraffin-embedded blocks. IHC staining was carried out as described previously [53], using the following primary antibodies: mouse anti-wild-type EGFR (M7298) and mouse anti-human CD44 mAb (M7082) (Agilent, Stockport, UK), anti-EGFRvIII (BS-2558R), rabbit pAb anti-Amphiregulin (GTX100986, InsightBiotech, London, UK), mouse anti-EGF (AHP767), mouse anti-TGFα (AHP284G) (Bio-Rad Antibodies, UK), mouse anti-Betacellulin (MAB2611) (Biotechne, Abingdon, UK), rabbit pAb anti-HBEGF (HPA053243), rabbit pAb anti-Epigen (HPA014420), and rabbit pAb anti-Epiregulin (HPA054373) (Merck, UK), and mouse anti-human CD133 (130-090-422) (Miltenyi Biotec, Surrey, UK).

### Scoring criteria

The immunostaining of the tumour sections were scored based on the percentage of tumour cells that had EGFR and/or ligands immunostaining (i.e. >5%, >10%, >20%, and >50%) and intensity of immunostaining (i.e. negative 0, weak 1+, moderate 2+ and strong 3+) and location (i.e. membrane, cytoplasm or nucleus of the cells). CD44 and CD133 sections were scored by the percentage of positive cells (0% to 50% and ≥50%) as low (^L^) and high (^H^), respectively. The immunostaining was scored by two independent observers without prior knowledge of the clinicopathological parameters and any disparity in scoring was resolved by simultaneous reassessment of the staining by both observers.

### Statistical analysis

The relationship between the expression levels of CSCs and response to treatment was determined by linear regression analysis and changes in the expression of CSCs in drug resistant variants were tested by Paired *T*-test analysis. The association between immunohistochemistry scores and patient clinicopathological data was assessed using Chi-Squared test (Pearson Chi-Square) and Fisher Exact Test. Kaplan-Meier survival plots were used to evaluate differences between groups by performing log rank-test. For univariate and multivariate analysis, the Cox regression model was used and *P* ≤ 0.05 was considered statistically significant. All statistical analyses were carried out using the PASW statistics 24 (SPPS Inc.).

## References

[1] Siegel RL, Miller KD, Jemal A (2018). Cancer statistics, 2018. CA Cancer J Clin.

[2] O’Brien CA, Pollett A, Gallinger S, Dick JE (2007). A human colon cancer cell capable of initiating tumour growth in immunodeficient mice. Nature.

[3] Dalerba P, Dylla SJ, Park IK, Liu R, Wang X, Cho RW, Hoey T, Gurney A, Huang EH, Simeone DM, Shelton AA, Parmiani G, Castelli C (2007). Phenotypic characterization of human colorectal cancer stem cells. Proc Natl Acad Sci U S A.

[4] Ricci-Vitiani L, Lombardi DG, Pilozzi E, Biffoni M, Todaro M, Peschle C, De Maria R (2007). Identification and expansion of human colon-cancer-initiating cells. Nature.

[5] Li C, Lee CJ, Simeone DM (2009). Identification of human pancreatic cancer stem cells. Methods Mol Biol.

[6] Trerotola M, Rathore S, Goel HL, Li J, Alberti S, Piantelli M, Adams D, Jiang Z, Languino LR (2010). CD133, Trop-2 and alpha2beta1 integrin surface receptors as markers of putative human prostate cancer stem cells. Am J Transl Res.

[7] Bussolati B, Dekel B, Azzarone B, Camussi G (2013). Human renal cancer stem cells. Cancer Lett.

[8] Wilson BJ, Schatton T, Frank MH, Frank NY (2011). Colorectal Cancer Stem Cells: Biology and Therapeutic Implications. Curr Colorectal Cancer Rep.

[9] Haraguchi N, Ohkuma M, Sakashita H, Matsuzaki S, Tanaka F, Mimori K, Kamohara Y, Inoue H, Mori M (2008). CD133+CD44+ population efficiently enriches colon cancer initiating cells. Ann Surg Oncol.

[10] Shmelkov SV, Butler JM, Hooper AT, Hormigo A, Kushner J, Milde T, St Clair R, Baljevic M, White I, Jin DK, Chadburn A, Murphy AJ, Valenzuela DM (2008). CD133 expression is not restricted to stem cells, and both CD133+ and CD133- metastatic colon cancer cells initiate tumors. J Clin Invest.

[11] Dittfeld C, Dietrich A, Peickert S, Hering S, Baumann M, Grade M, Ried T, Kunz-Schughart LA (2009). CD133 expression is not selective for tumour-initiating or radioresistant cell populations in the CRC cell lines HCT-116. Radiotherapy and Oncology.

[12] Du L, Wang H, He L, Zhang J, Ni B, Wang X, Jin H, Cahuzac N, Mehrpour M, Lu Y, Chen Q (2008). CD44 is of functional importance for colorectal cancer stem cells. Clin Cancer Res.

[13] Elsaba TMA, Martinez-Pomares L, Robins AR, Crook S, Seth R, Jackson D, McCart A, Silver AR, Tomlinson IP, Ilyas M (2010). The Stem Cell Marker CD133 Associates with Enhanced Colony Formation and Cell Motility in Colorectal Cancer. PLoS One.

[14] Fang DD, Kim YJ, Lee CN, Aggarwal S, McKinnon K, Mesmer D, Norton J, Birse CE, He T, Ruben SM, Moore PA (2010). Expansion of CD133+ colon cancer cultures retaining stem cell properties to enable cancer stem cell target discovery. Br J Cancer.

[15] Leng Z, Xia Q, Chen J, Li Y, Xu J, Zhao E, Zheng H, Ai W, Dong J (2018). Lgr5+CD44+EpCAM+ Strictly Defines Cancer Stem Cells in Human Colorectal Cancer. Cell Physiol Biochem.

[16] Pitule P, Cedikova M, Daum O, Vojtisek J, Vycital O, Hosek P, Treska V, Hes O, Kralickova M, Liska V (2014). Immunohistochemical detection of cancer stem cell related markers CD44 and CD133 in metastatic colorectal cancer patients. Biomed Res Int.

[17] Du L, Rao G, Wang H, Li B, Tian W, Cui J, He L, Laffin B, Tian X, Hao C, Liu H, Sun X, Zhu Y (2013). CD44-positive cancer stem cells expressing cellular prion protein contribute to metastatic capacity in colorectal cancer. Cancer Res.

[18] Khelwatty SA, Essapen S, Seddon AM, Modjtahedi H (2013). Prognostic significance and targeting of HER family in colorectal cancer. Front Biosci (Landmark Ed).

[19] Wong SF (2005). Cetuximab: an epidermal growth factor receptor monoclonal antibody for the treatment of colorectal cancer. Clin Ther.

[20] Shih T, Lindley C (2006). Bevacizumab: an angiogenesis inhibitor for the treatment of solid malignancies. Clin Ther.

[21] Wu M, Rivkin A, Pham T (2008). Panitumumab: Human monoclonal antibody against epidermal growth factor receptors for the treatment of metastatic colorectal cancer. Clinical Therapeutics.

[22] Chu E (2012). An update on the current and emerging targeted agents in metastatic colorectal cancer. Clin Colorectal Cancer.

[23] Tol J, Koopman M, Cats A, Rodenburg CJ, Creemers GJ, Schrama JG, Erdkamp FL, Vos AH, van Groeningen CJ, Sinnige HA, Richel DJ, Voest EE, Dijkstra JR (2009). Chemotherapy, bevacizumab, and cetuximab in metastatic colorectal cancer. N Engl J Med.

[24] Amado RG, Wolf M, Peeters M, Van Cutsem E, Siena S, Freeman DJ, Juan T, Sikorski R, Suggs S, Radinsky R, Patterson SD, Chang DD (2008). Wild-type KRAS is required for panitumumab efficacy in patients with metastatic colorectal cancer. J Clin Oncol.

[25] Stintzing S, Fischer von Weikersthal L, Decker T, Vehling-Kaiser U, Jager E, Heintges T, Stoll C, Giessen C, Modest DP, Neumann J, Jung A, Kirchner T, Scheithauer W (2012). FOLFIRI plus cetuximab versus FOLFIRI plus bevacizumab as first-line treatment for patients with metastatic colorectal cancer-subgroup analysis of patients with KRAS: mutated tumours in the randomised German AIO study KRK-0306. Ann Oncol.

[26] Khelwatty SA, Essapen S, Seddon AM, Modjtahedi H (2011). Growth response of human colorectal tumour cell lines to treatment with afatinib (BIBW2992), an irreversible erbB family blocker, and its association with expression of HER family members. Int J Oncol.

[27] Khelwatty SA, Essapen S, Seddon AM, Fan Z, Modjtahedi H (2015). Acquired resistance to anti-EGFR mAb ICR62 in cancer cells is accompanied by an increased EGFR expression, HER-2/HER-3 signalling and sensitivity to pan HER blockers. Br J Cancer.

[28] Papailiou J, Bramis KJ, Gazouli M, Theodoropoulos G (2011). Stem cells in colon cancer. A new era in cancer theory begins. Int J Colorectal Dis.

[29] Bapat SA (2010). Human ovarian cancer stem cells. Reproduction.

[30] Choi D, Lee HW, Hur KY, Kim JJ, Park GS, Jang SH, Song YS, Jang KS, Paik SS (2009). Cancer stem cell markers CD133 and CD24 correlate with invasiveness and differentiation in colorectal adenocarcinoma. World J Gastroenterol.

[31] Horst D, Kriegl L, Engel J, Kirchner T, Jung A (2008). CD133 expression is an independent prognostic marker for low survival in colorectal cancer. British Journal of Cancer.

[32] Horst D, Kriegl L, Engel J, Kirchner T, Jung A (2009). Prognostic significance of the cancer stem cell markers CD133, CD44, and CD166 in colorectal cancer. Cancer Invest.

[33] Galizia G, Gemei M, Del Vecchio L, Zamboli A, Di Noto R, Mirabelli P, Salvatore F, Castellano P, Orditura M, De Vita F, Pinto M, Pignatelli C, Lieto E (2012). Combined CD133/CD44 expression as a prognostic indicator of disease-free survival in patients with colorectal cancer. Arch Surg.

[34] Lugli A, Iezzi G, Hostettler I, Muraro MG, Mele V, Tornillo L, Carafa V, Spagnoli G, Terracciano L, Zlobec I (2010). Prognostic impact of the expression of putative cancer stem cell markers CD133, CD166, CD44s, EpCAM, and ALDH1 in colorectal cancer. Br J Cancer.

[35] Jing F, Kim HJ, Kim CH, Kim YJ, Lee JH, Kim HR (2015). Colon cancer stem cell markers CD44 and CD133 in patients with colorectal cancer and synchronous hepatic metastases. Int J Oncol.

[36] Huang R, Mo D, Wu J, Ai H, Lu Y (2018). CD133 expression correlates with clinicopathologic features and poor prognosis of colorectal cancer patients: An updated meta-analysis of 37 studies. Medicine (Baltimore).

[37] Zhao Y, Peng J, Zhang E, Jiang N, Li J, Zhang Q, Zhang X, Niu Y (2016). CD133 expression may be useful as a prognostic indicator in colorectal cancer, a tool for optimizing therapy and supportive evidence for the cancer stem cell hypothesis: a meta-analysis. Oncotarget.

[38] Khelwatty S, Essapen S, Bagwan I, Green M, Seddon A, Modjtahedi H (2017). The impact of co-expression of wild-type EGFR and its ligands determined by immunohistochemistry for response to treatment with cetuximab in patients with metastatic colorectal cancer. Oncotarget.

[39] Peciak J, Stec WJ, Treda C, Ksiazkiewicz M, Janik K, Popeda M, Smolarz M, Rosiak K, Hulas-Bigoszewska K, Och W, Rieske P, Stoczynska-Fidelus E (2017). Low Incidence along with Low mRNA Levels of EGFR(vIII) in Prostate and Colorectal Cancers Compared to Glioblastoma. J Cancer.

[40] Heimberger AB, Hlatky R, Suki D, Yang D, Weinberg J, Gilbert M, Sawaya R, Aldape K (2005). Prognostic effect of epidermal growth factor receptor and EGFRvIII in glioblastoma multiforme patients. Clinical Cancer Research.

[41] Yun S, Kwak Y, Nam SK, Seo AN, Oh HK, Kim DW, Kang SB, Lee HS (2018). Ligand-Independent Epidermal Growth Factor Receptor Overexpression Correlates with Poor Prognosis in Colorectal Cancer. Cancer Res Treat.

[42] Alvarez K, Orellana P, Villarroel C, Contreras L, Kawachi H, Kobayashi M, Wielandt AM, De la Fuente M, Trivino JC, Kronberg U, Carvallo P, Lopez-Kostner F (2017). EGFR pathway subgroups in Chilean colorectal cancer patients, detected by mutational and expression profiles, associated to different clinicopathological features. Tumour Biol.

[43] Koustas E, Karamouzis MV, Mihailidou C, Schizas D, Papavassiliou AG (2017). Co-targeting of EGFR and autophagy signaling is an emerging treatment strategy in metastatic colorectal cancer. Cancer Lett.

[44] Pietrantonio F, Vernieri C, Siravegna G, Mennitto A, Berenato R, Perrone F, Gloghini A, Tamborini E, Lonardi S, Morano F, Picciani B, Busico A, Volpi CC (2017). Heterogeneity of Acquired Resistance to Anti-EGFR Monoclonal Antibodies in Patients with Metastatic Colorectal Cancer. Clin Cancer Res.

[45] Ohchi T, Akagi Y, Kinugasa T, Kakuma T, Kawahara A, Sasatomi T, Gotanda Y, Yamaguchi K, Tanaka N, Ishibashi Y, Miyamoto S, Kage M, Shirouzu K (2012). Amphiregulin is a prognostic factor in colorectal cancer. Anticancer Res.

[46] Kuramochi H, Nakajima G, Kaneko Y, Nakamura A, Inoue Y, Yamamoto M, Hayashi K (2012). Amphiregulin and Epiregulin mRNA expression in primary colorectal cancer and corresponding liver metastases. BMC Cancer.

[47] Li XD, Miao SY, Wang GL, Yang L, Shu YQ, Yin YM (2010). Amphiregulin and epiregulin expression in colorectal carcinoma and the correlation with clinicopathological characteristics. Onkologie.

[48] Jacobs B, De Roock W, Piessevaux H, Van Oirbeek R, Biesmans B, De Schutter J, Fieuws S, Vandesompele J, Peeters M, Van Laethem JL, Humblet Y, Penault-Llorca F, De Hertogh G (2009). Amphiregulin and epiregulin mRNA expression in primary tumors predicts outcome in metastatic colorectal cancer treated with cetuximab. J Clin Oncol.

[49] Yamada M, Ichikawa Y, Yamagishi S, Momiyama N, Ota M, Fujii S, Tanaka K, Togo S, Ohki S, Shimada H (2008). Amphiregulin is a promising prognostic marker for liver metastases of colorectal cancer. Clin Cancer Res.

[50] Yun S, Koh J, Nam SK, Park JO, Lee SM, Lee K, Lee KS, Ahn SH, Park DJ, Kim HH, Choe G, Kim WH, Lee HS (2018). Clinical significance of overexpression of NRG1 and its receptors, HER3 and HER4, in gastric cancer patients. Gastric Cancer.

[51] Wang J, Yin J, Yang Q, Ding F, Chen X, Li B, Tian X (2016). Human epidermal growth factor receptor 4 (HER4) is a favorable prognostic marker of breast cancer: a systematic review and meta-analysis. Oncotarget.

[52] Fujiwara S, Hung M, Yamamoto-Ibusuk CM, Yamamoto Y, Yamamoto S, Tomiguchi M, Takeshita T, Hayashi M, Sueta A, Iwase H (2014). The localization of HER4 intracellular domain and expression of its alternately-spliced isoforms have prognostic significance in ER+ HER2- breast cancer. Oncotarget.

[53] Khelwatty SA, Essapen S, Bagwan I, Green M, Seddon AM, Modjtahedi H (2014). Co-expression of HER family members in patients with Dukes’ C and D colon cancer and their impacts on patient prognosis and survival. PLoS One.

[54] Nautiyal J, Du J, Yu Y, Kanwar SS, Levi E, Majumdar AP (2012). EGFR regulation of colon cancer stem-like cells during aging and in response to the colonic carcinogen dimethylhydrazine. Am J Physiol Gastrointest Liver Physiol.

[55] Piao LS, Hur W, Kim TK, Hong SW, Kim SW, Choi JE, Sung PS, Song MJ, Lee BC, Hwang D, Yoon SK (2012). CD133+ liver cancer stem cells modulate radioresistance in human hepatocellular carcinoma. Cancer Lett.

[56] Croker AK, Allan AL (2012). Inhibition of aldehyde dehydrogenase (ALDH) activity reduces chemotherapy and radiation resistance of stem-like ALDHhiCD44(+) human breast cancer cells. Breast Cancer Res Treat.

[57] Alvero AB, Chen R, Fu HH, Montagna M, Schwartz PE, Rutherford T, Silasi DA, Steffensen KD, Waldstrom M, Visintin I, Mor G (2009). Molecular phenotyping of human ovarian cancer stem cells unravels the mechanisms for repair and chemoresistance. Cell Cycle.

[58] Casagrande F, Cocco E, Bellone S, Richter CE, Bellone M, Todeschini P, Siegel E, Varughese J, Arin-Silasi D, Azodi M, Rutherford TJ, Pecorelli S, Schwartz PE (2011). Eradication of chemotherapy-resistant CD44+ human ovarian cancer stem cells in mice by intraperitoneal administration of Clostridium perfringens enterotoxin. Cancer.

[59] Bray F, Ferlay J, Soerjomataram I, Siegel RL, Torre LA, Jemal A (2018). Global cancer statistics 2018: GLOBOCAN estimates of incidence and mortality worldwide for 36 cancers in 185 countries. CA Cancer J Clin.

[60] Cunningham MP, Thomas H, Fan Z, Modjtahedi H (2006). Responses of human colorectal tumor cells to treatment with the anti-epidermal growth factor receptor monoclonal antibody ICR62 used alone and in combination with the EGFR tyrosine kinase inhibitor gefitinib. Cancer Research.

[61] Modjtahedi H, Styles JM, Dean CJ (1993). The human EGF receptor as a target for cancer therapy: six new rat mAbs against the receptor on the breast carcinoma MDA-MB 468. British Journal of Cancer.

